# Performance Assessment of Two Whole-Lake Acoustic Positional Telemetry Systems - Is Reality Mining of Free-Ranging Aquatic Animals Technologically Possible?

**DOI:** 10.1371/journal.pone.0126534

**Published:** 2015-05-22

**Authors:** Henrik Baktoft, Petr Zajicek, Thomas Klefoth, Jon C. Svendsen, Lene Jacobsen, Martin Wæver Pedersen, David March Morla, Christian Skov, Shinnosuke Nakayama, Robert Arlinghaus

**Affiliations:** 1 National Institute of Aquatic Resources, Technical University of Denmark, Silkeborg, Denmark; 2 Department of Biology and Ecology of Fishes, Leibniz-Institute of Freshwater Ecology and Inland Fisheries, Berlin, Germany; 3 Interdisciplinary Centre of Marine and Environmental Research, University of Porto, Porto, Portugal; 4 National Institute of Aquatic Resources, Technical University of Denmark, Copenhagen, Denmark; 5 Mediterranean Institute for Advanced Studies, University of the Balearic Islands, Esporles, Islas Baleares, Spain; 6 Division of Integrative Fisheries Management, Faculty of Life Sciences, Humboldt Universität zu Berlin, Berlin, Germany; Pacific Northwest National Laboratory, UNITED STATES

## Abstract

Acoustic positional telemetry systems (APTs) represent a novel approach to study the behaviour of free ranging aquatic animals in the wild at unprecedented detail. System manufactures promise remarkably high temporal and spatial resolution. However, the performance of APTs has rarely been rigorously tested at the level of entire ecosystems. Moreover, the effect of habitat structure on system performance has only been poorly documented. Two APTs were deployed to cover two small lakes and a series of standardized stationary tests were conducted to assess system performance. Furthermore, a number of tow tests were conducted to simulate moving fish. Based on these data, we quantified system performance in terms of data yield, accuracy and precision as a function of structural complexity in relation to vegetation. Mean data yield of the two systems was 40 % (Lake1) and 60 % (Lake2). Average system accuracy (acc) and precision (prec) were Lake1: acc = 3.1 m, prec = 1.1 m; Lake2: acc = 1.0 m, prec = 0.2 m. System performance was negatively affected by structural complexity, i.e., open water habitats yielded far better performance than structurally complex vegetated habitats. Post-processing greatly improved data quality, and sub-meter accuracy and precision were, on average, regularly achieved in Lake2 but remained the exception in the larger and structurally more complex Lake1. Moving transmitters were tracked well by both systems. Whereas overestimation of moved distance is inevitable for stationary transmitters due to accumulation of small tracking errors, moving transmitters can result in both over- and underestimation of distances depending on circumstances. Both deployed APTs were capable of providing high resolution positional data at the scale of entire lakes and are suitable systems to mine the reality of free ranging fish in their natural environment. This opens important opportunities to advance several fields of study such as movement ecology and animal social networks in the wild. It is recommended that thorough performance tests are conducted in any study utilizing APTs. The APTs tested here appear best suited for studies in structurally simple ecosystems or for studying pelagic species. In such situations, the data quality provided by the APTs is exceptionally high.

## Introduction

Animal behaviour drives many ecological and evolutionary processes in both terrestrial and aquatic environments. In aquatic ecosystems, fish behaviour can for example influence nutrient dynamics and trophic status [1] and affect the diversity and stability of communities [2]. From an evolutionary point of view, behaviour can compensate for alternative phenotypic traits [3] and may influence whether a novel trait spreads in a population [4]. The importance of behaviour is highlighted by the recent emphasis on fisheries-induced evolution where direct selection on behaviour can play a significant role in life-history evolution [5–7]. Correspondingly, behaviour is increasingly integrated into ecological [8] and eco-evolutionary models [9]. However, furthering our understanding of the relationship of individual animal behaviour to population ecology and evolution in aquatic environments is often severely constrained by technical limitations that make high resolution observations in the wild difficult.

Biotelemetry constitutes a versatile approach to study fish behaviour in natural settings [10–12]. Studies using biotelemetry in aquatic environments range from aquaculture-based studies in confined environments to assessments of fish behaviour in lakes, streams, lagoons and even across oceans (for reviews see, e.g., [11,13–17]). However, due to technological limitations little is known about fine scale (both temporal and spatial) behaviour of fishes in their natural environment at the scale of entire ecosystems [12], although examples are increasingly appearing in the literature [18–20].

Following the introduction of acoustic-based positional telemetry to aquatic ecology in the 1970s [21], refinements to the methodology have enabled high resolution, near real-time tracking of large numbers of tagged individuals *in situ* [22–28]. These technologies promise exciting possibilities to quantify fine scale behaviour of free ranging fish by turning study sites into field-laboratories that allow “mining the reality” of the animal’s life in space and time [12]. In recent years, a number of studies utilizing acoustic positional telemetry (APT) systems have been published (e.g., [18–20,27–33]). However, rigorous performance assessments of APT systems in relation to the validity and reliability of the data at the level of entire ecosystems are still relatively scarce (but see [25–27,34–39]). Given the many technological challenges that await when running long-term (i.e., several months or even years) acoustic telemetry studies in the wild (e.g., [26,34,35,40]), the rigorous performance testing of APT systems is a prerequisite for generating faith in the data.

High resolution APT systems are based on transmitters emitting acoustic signals that propagate through water and are ultimately registered by a network of hydrophones positioned at precisely known locations. The difference in time of arrival of the transmitter signals at various hydrophones is used in a positioning algorithm based on hyperbolic tri- or multilateration. Therefore, any factor influencing or interfering with speed of sound and propagation of acoustic signals in water will affect the performance of APT systems [25,26,34,35]. In particular, strong thermoclines, vegetation or other obstacles to acoustic signal propagation may limit the applicability of high resolution APT systems. This will likely be ecosystem-specific requiring specific calibration to local conditions [35]. Some manufactures of APT systems provide a set of data quality metrics computed for each calculated position (e.g., [22,41]) that can be used for filtering [23,26,37,38]. However, even though this process will reduce errors, no field studies involving known-location transmitters have been able to fully eliminate spatial biases [25–27,34,35].

Available APT systems utilize different technologies to encode and relay transmitter ID and associated information from optional sensors in the acoustic signal. Furthermore, hardware and post-processing routines differ between commercial APT systems. Additionally, in some systems (e.g., Vemco), the manufacturers retain responsibility for the positioning and filtering of positions, whereas the selected supplier for this study, Lotek Wireless, allows end-users to develop own post-processing routines. Therefore, although all APT systems are ultimately based on hyperbolic tri- or multilateration (e.g., [22,42]), error sources and system performance can vary among suppliers. A few studies evaluating APT system performance have reported average positional accuracies ranging between 1.6 and 10.7 m (e.g., [25,34,35]) suggesting that fine-scale positional telemetry is possible in the wild, although the often promised sub-meter accuracy [42] may not be attainable in every situation.

The commercial supplier Lotek Wireless provides the MAP system, which exists in both a wired and a wireless version. Both versions are unique because they are based on a code-division-multiple-access (CDMA) encoding scheme that is robust against code collision and noise interference and allows several thousand individual transmitters to transmit on the same acoustic frequency [22]. Previous studies using Lotek MAP equipment reported that sub-meter accuracy is achievable using CDMA systems [22,23,42], but the studies did not provide rigorous replicated tests in support of the accuracy statements or used state-based model predictions of positions based on a known deviation from true positions [27]. Overall, it remains unclear how often sub-meter accuracy is achieved and whether accuracy varies with habitat structure in natural ecosystems. Any effect of habitat structure on the positional data could have important implications for data interpretation and study conclusions. Recently, [26] tested the performance of a wireless Lotek MAP system deployed at artificial marine reefs and reported average positional accuracy of 2 m. Furthermore, the same authors reported satisfactory system robustness against potential user-induced error sources related to sound speed calculations and precise location of hydrophone position. It is so far unclear how these experiences translate from saltwater to freshwater environments at the scale of entire lakes. As many lakes and rivers are eutrophic and may have high phytoplankton abundance, signal attenuation of acoustic transmitters could be more pronounced in freshwater than in nutrient-poorer marine environments or reservoirs [27], which might affect system performance.

The objective of this study was to examine the potential of whole-lake APT systems for detailed studies of free-ranging wild aquatic animals. To this end, we tested the performance of two CDMA-based APT systems from Lotek Wireless deployed in two different European shallow lakes. We focused particularly on the system specific performance of the two APT systems in densely structured littoral zones and non-structured pelagic zones replicating tag exposure within lakes and confining statistical analysis to data sets within each lake. This was done because many temperate fish species are known to depend on structured habitats. Our study provides the first test of APT systems in relation to habitat structure at the level of whole ecosystems.

## Materials and Methods

### Study sites

**Fig 1:**
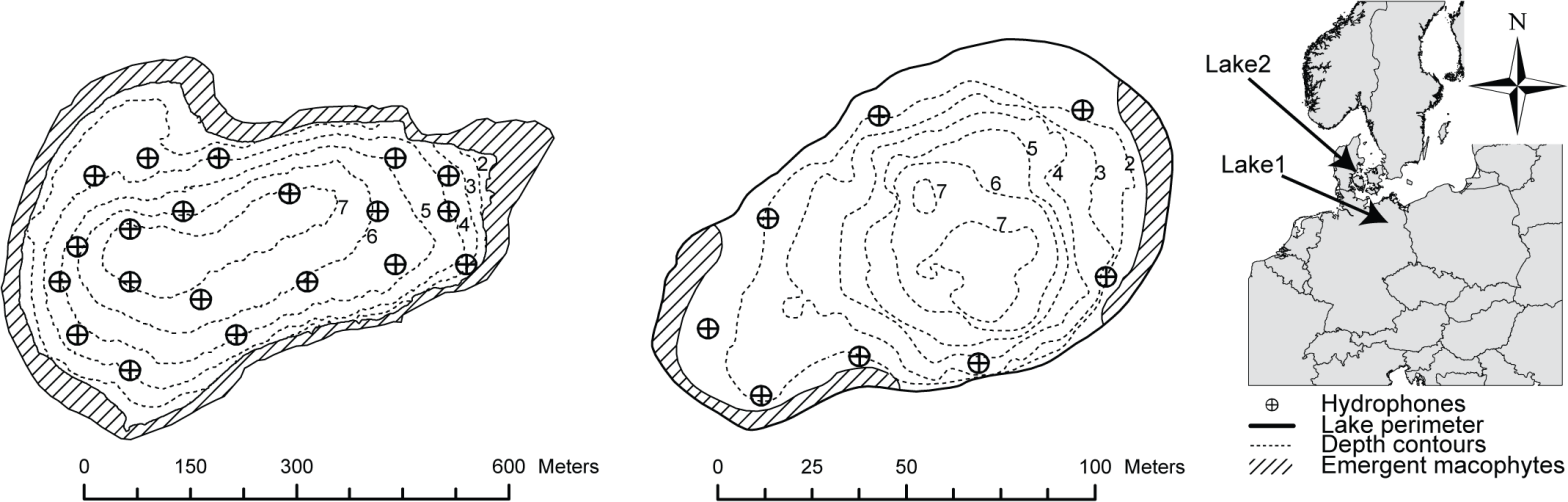
Overview of the two study lakes (top: Lake1, bottom: Lake2).

The study was conducted in two shallow lowland lakes located in Germany and Denmark, each equipped with an APT system based on the CDMA technology provided by Lotek Wireless (Fig 1; Table 1). Both systems were deployed to cover the entire lake. Lake1 (Kleiner Döllnsee, 52°59´ N, 13°34´ E, Germany, 25 ha, maximum depth 8 m) is a slightly eutrophic lake with well-developed submerged (primarily *Potamogeton* spp.) and emergent vegetation along the shores (primarily *Phragmites* and *Typha* spp.). Lake2 (Lake Gosmer, 55°55´ N, 10°10´ E, Denmark, 1 ha, maximum depth 8 m) is a hyper-eutrophic lake in which submerged vegetation is scarce, whereas emergent vegetation (*Typha latifolia*) is widespread along the shore. For further details on the study lakes, see previous studies [31,43].

**Table 1:** Lake specific summaries.

	Lake1	Lake2
	Kleiner Döllnsee	Gosmer
Area (km^2^)	0.25	0.01
Max depth (m)	7.6	8
Mean depth (m)	4.1	2.9
Emergent macrohyte coverage (%)	20	10
Submerged macrohyte coverage (%)	50	1
Mean total phosphorus (μg/L)	29	37
Mean Chlorohyll A (μg/L)	9.9	43
Mean Secchi depth (m)	3.2	1.2
Stratified	Yes	Yes
CDMA version	Wireless (WHS3050)	Wired (MAP_600)
Number of hydrophones	20	8
Mean hydrophone dist (m)	272	65
Min—Max hydrophone distance (m)	48–576	23–115

Lake1 was equipped with a wireless version of the Lotek MAP System (WHS 3050; 200 kHz; Lotek Wireless Inc., Newmarket, Ontario, Canada) consisting of 20 wireless receivers with integrated dataloggers (henceforth referred to as hydrophones), clock-synchronized during data processing based on signals from beacon transmitters attached to each hydrophone [26]. Hydrophones were attached to PVC-tubes sliding over steel tubes, which were driven into the lake bottom and positioned approximately two meters below surface (see [44] for details). Lake2 was equipped with a cabled version of the Lotek MAP System (MAP600; 200 kHz; Lotek Wireless Inc., Newmarket, Ontario, Canada) consisting of eight cabled hydrophones connected to and clock-synchronised by a single onshore datalogger. Precise geographic positions of all hydrophones were obtained using a differential GPS-unit (determined to ± 0.2 m, Trimble GeoXH, Sunnyvale, California, USA). Hydrophones were positioned approximately one meter below surface and attached to steel tubes that were driven into the lake bottom. While the hardware setup in the two lakes was dissimilar, both systems utilized comparable technology (e.g., CDMA technology and identical transmitter frequencies) and post-processing algorithms for calculating positions of the transmitters. The specific layout of the hydrophone arrays in both lakes were based on manufacturer recommendations provided after in-field study site assessments combined with theoretical performance calculations using vendor supplied software (BioMAP version 2.1.12.1; Lotek Wireless), range testing and experience from previous studies. In both lakes, the vertical positions of the hydrophones were chosen to ensure that fish and hydrophones were in the same part of the water column in periods of stratification during which anoxia potentially developed in the hypolimnion.

Transmitters were based on coded signals proprietary to the manufacturer (Lotek Wireless). Signals emitted from transmitters were detected by hydrophones, stored and downloaded to a portable computer for subsequent analysis using proprietary software (Lake1: Asynchronus Logger Positioning System, ALPS, version 2.22; Lake2: BioMAP version 2.1.12.1; both from Lotek Wireless). If a signal was detected by at least three hydrophones, a 2D position was calculated based on differences in time of arrival at each hydrophone using hyperbolic tri- or multilateration techniques [22,42,45]. The transmitters used in Lake1 were combined acoustic-radio transmitters of the model CH-TP16 (burst interval 9.2 s, every third signal was relayed as a radio signal and not detectable by the APT), and in Lake2 acoustic transmitters of model MAP6_2 were used (burst interval 2.56 s). Although being different models that might differ in performance, the transmitters were technically comparable and used identical frequency (200 kHz) and coding technology (CDMA). All tags were unused at the onset of this study and originally purchased for biological studies on fish behaviour. Besides the identification code the transmitters used in Lake1 relayed information from integrated temperature and pressure sensors, enabling calculation of transmitter depth. The temperature data were not used in the present study.

### Performance assessment of stationary transmitters to simulate non-moving fish

To assess data yield, accuracy and precision of the two systems, a number of stationary tests with known-location transmitters were conducted following a random stratified survey design, with stratification based on mesohabitat categories present in each of the two lakes (Lake1: n = 90; Lake2: n = 50). All tests were conducted during three consecutive days in September 2010 (Lake1) and November 2010 (Lake2). During the tests the water column in both lakes were fully mixed (i.e., no thermocline present) and mean water temperatures were 17.1°C in Lake1 and 5.4°C in Lake2. Transmitters were moored at known positions (determined to ± 0.2 m using a differential GPS-unit (DGPS), Trimble GeoXH, Sunnyvale, California, USA) naturally covering different habitat types (strata), allowing the transmitters to emit several hundred signals during each trial. Mean duration of tests were 178 minutes (range 55–824) in Lake1 and 24 minutes (range 16–49) in Lake2. Transmitters were deployed to mimic positions of a stationary fish, by attaching them to a line (diameter 5 mm), held in place by a heavy weight and kept vertical by a float. Up to four transmitters were attached to each line at known distances from the water surface. Each deployment of a transmitter was treated as a sampling unit in subsequent analyses, yielding a total of 155 (Lake1) and 123 (Lake2) trials. Transmitters were fixed horizontally on the line to mimic the orientation of a transmitter implanted in a fish. Habitat structure and complexity at the sites of deployment were categorized based on depth as well as type and quantity of the vegetation in immediate vicinity of the transmitter (radius of 1 m) assessed by eye and underwater cameras. Habitat categories included: dense submerged macrophytes (SD), above submerged macrophytes (SA), shallow open water (OS), deep open water (OD), loose emergent macrophytes (EL) and dense emergent macrophytes (ED). All habitat categories were present in Lake1, whereas Lake2 did not contain sufficient submerged macrophytes to test categories SA and SD.

System efficiency (i.e., data yield) was defined as the proportion of emitted signals resulting in an estimated position. Accuracy of calculated positions was defined as deviation from true position estimated as the Euclidian distance between estimated and true position based on the DGPS measurements. For each stationary test, accuracy was calculated as the mean of these distances generated during the deployment time. Precision was defined as the variability of those distances and was calculated for each stationary test as the standard deviation of the estimated accuracy. Efficiency, accuracy and precision were compared statistically for an effect of habitat structural complexity, treating each transmitter deployment as a sampling unit.

Any inaccuracy of estimated positions will induce apparent false movement when the distances between positions are summed in actual field applications, for example when deriving estimates of total moved distances per unit time. To assess the degree of system-generated false movement rates, including an effect of habitat structural complexity, false movement rates were calculated as the mean distance between consecutive positions for each stationary trial. The resulting trial means were statistically compared across habitat types and extrapolated for comparison with tow tests.

### Performance assessment of tow tests to simulate swimming fish

Stationary tests are inadequate to assess system performance for moving animals. Therefore, several tow tests (Lake1: n = 6, mean duration = 35.5 minutes; Lake2: n = 22, mean duration = 3 minutes) were conducted in each lake to mimic trajectories of fish circling and crossing the lake. These tests were performed during the same periods as the stationary tests. Three transmitters were attached at different depths within the upper 1.5 meter to a solid vertical rod mounted on a boat propelled by an electric motor yielding 18 and 66 trials, respectively. Mean speed of the boat was 0.58 m s^-1^ (s.d. = 0.16) in Lake1 and 0.43 m s^-1^ (s.d. = 0.066) in Lake2. True trajectories were determined (± 0.2 m, sampling rate = 1 Hz) using a DGPS (Trimble GeoXH, Sunnyvale, California, USA) positioned directly above the transmitters. System efficiency and precision for moving transmitters was assessed following the protocol from the stationary tests. Accuracy calculations were based on minimum distances between calculated positions and the DGPS trajectory. Additionally, total track length of the trajectories obtained from the APTs was calculated and compared to true track length obtained from DGPS.

### Reliability of pressure sensors to assess fish depth

For each trial in Lake1, the distance from the transmitter to the water surface (i.e., transmitter depth), was measured, thereby allowing an assessment of the accuracy and precision of integrated pressure sensors. Pressure sensors relay unitless values between 0 and 100, representing increasing pressure as part of the coded signal. Transmitter outputs were rescaled to depth units (m) and used to assess accuracy and precision of the third (vertical) dimension.

### Raw data handling and filtering

Raw positions generated from the positioning software contained outliers and tracking error. To remove obvious outliers, an initial filtering based on positional quality metrics provided with each position by the Lotek Wireless software (i.e., Dilution Of Precision (DOP), Condition Number (CN) and Reliability Number (RN)) was applied as suggested by [22] and [26]. We initially used the following threshold values for exclusion of a given position: DOP < 10, CN < 10 and RN > 0. Additionally, in Lake1 positions outside the lake perimeter were deleted. The resulting data are henceforth referred to as raw positions. To further reduce the effect of system-induced false positions, the raw data were further filtered using stricter quality metric values (i.e., DOP < 1, CN < 10 and RN > 1), which should facilitate high quality data with limited error [26,27] but also reduces the number of positions. The remaining data were subsequently smoothed using a Hidden Markov Model (HMM) with a t-distributed observation noise following [46,47]. The HMM estimates a two-dimensional probability distribution for each position using information from the focal as well as the prior and following positions. Most probable positions were obtained as means in these probability distributions. The HMM did not reduce the number of positions. The data resulting from the stricter filtering and subsequent HMM-smoothing are henceforth referred to as filtered positions.

**Fig 2:**
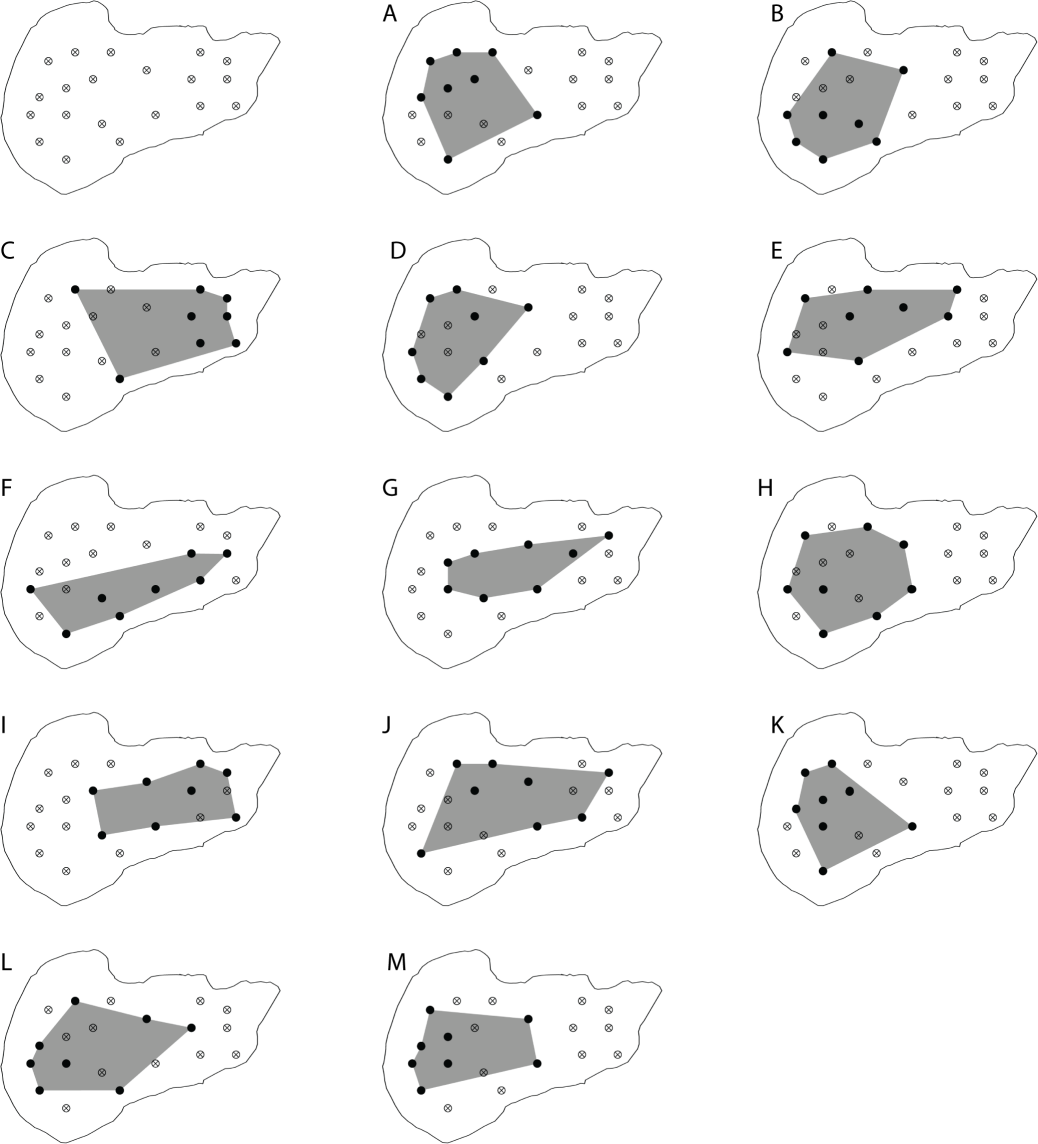
Defined sub-arrays in Lake1. Entire array (upper left) and the thirteen sub-arrays (A-M) defined in Lake1 to facilitate lake-wide positioning. Black solid circles indicate arrays included in a given sub-array. The footprint of each sub-array is indicated as grey areas to help visualizing array layout.

By employing the proprietary software by Lotek Wireless for both the cabled and wireless versions available at time the study was conducted, positions could only be calculated using a maximum of eight hydrophones forming an array. To facilitate lake-wide positioning in Lake1 (20 hydrophones in total), 13 ad hoc 8-hydrophone arrays were defined and processed separately (Fig 2). As a result, for each given signal potentially received by multiple hydrophones, multiple positions (theoretically up to 13) could be generated if the same signal was recorded by at least three hydrophones in different arrays. Furthermore, due to inevitable clock drift in the wireless telemetry system, the arrays were not completely in time synchrony with each other. Therefore, the timestamps of multiple calculated positions from the same signal could differ slightly. To address this, raw positions from Lake1 were pre-processed to ensure that any multiple calculated positions from each emitted signal had identical timestamps prior to applying the HMM. As a result, the final data produced by the HMM contained only one position per timestamp representing the most probable position.

### Statistical analysis

For the stationary tests, effects of habitat type and complexity on data yield, accuracy and precision of raw and filtered data within each lake were analysed by fitting general linear models (LM) using generalized least squares [48]. In each test, habitat type was entered as fixed factor, and data efficiency, accuracy or precision as dependent variables. Analyses were completed for each study lake separately. A total of six models were fitted, one for each of the dependent variables in each lake. To assess the significance of differences between habitat types pair-wise post-hoc comparison tests were performed by sequentially shifting the baseline in the LM models. Habitat categories with less than four trials yielding positions were excluded from analyses of accuracy and precision. Prior to fitting the models, efficiency was arcsin-transformed, and both accuracy and precision were log(y + 0.1) transformed to meet model assumptions of normality following [49]. A variance structure was included to allow for heterogeneity of variances between habitat categories when this significantly improved model fit [48].

The relationship between true transmitter depth and depth reported by the transmitters was analysed by fitting a LM. Trial means of reported depth was entered as explanatory variable and true transmitter depth as dependent variable. All statistical analyses were done in R version 3.0.2 [50] using the nlme 3.1–11 package [51].

### Ethics statement

No animals were used in this study, thus no permits or approvals were applicable. Both lakes are privately owned and access was granted by the land owners.

## Results

### Stationary tests

**Fig 3:**
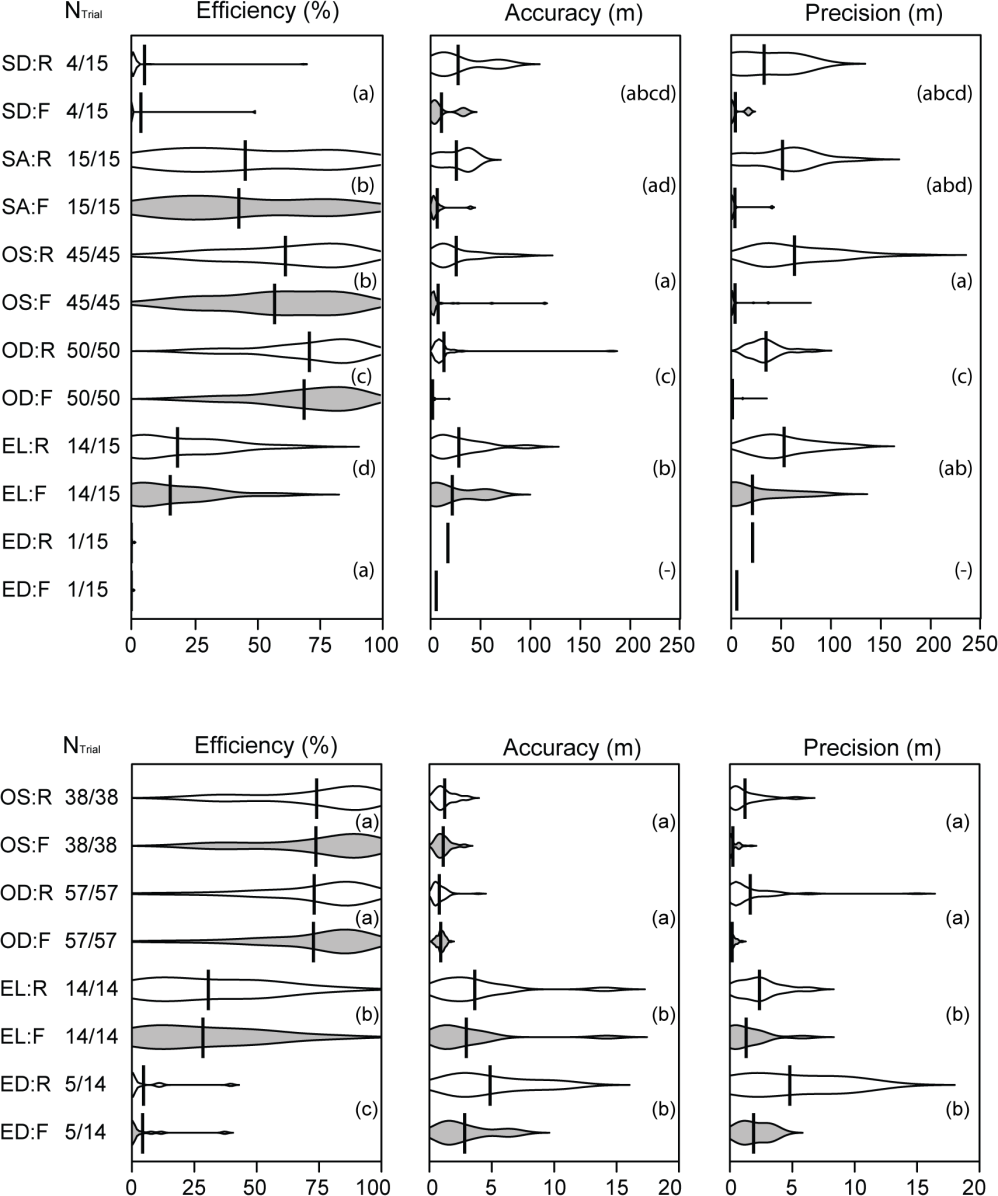
Results from stationary performance tests. Distributions of efficiency, accuracy and precision from Lake1 (upper row) and Lake2 (bottom row) in the six different habitat categories (SD = dense submerged macrophytes; SA = above submerged macrophytes; OS = shallow open water; OD = deep open water; EL = loose emergent macrophytes; ED = dense emergent macrophytes). R and F denote raw (white) and filtered (grey) data, respectively. Shape of each distribution indicates data point density. Mean values are given by solid vertical bars. N_Trial_ indicates number of trials yielding positions / number of trials in each habitat type. Dissimilar letters inside each panel indicate groups of non-significant (p > 0.05) differences obtained from pair-wise post-hoc comparisons using filtered data. Note scale differences between the two lakes.

System performance in both lakes was strongly affected by habitat type (p < 0.01 in all models; Table 2; Fig 3). In both lakes, best system performance was achieved in the structurally simple pelagic habitat (average values obtained from the statistical models (back-transformed to original scale) ± standard deviation (s.d.) in brackets; note s.d. are non-symmetrical due to back-transformation and hence are presented as ranges; Lake1: data yield = 70.8% (49.3–88.4), accuracy = 1.9 m (1.2–3.0), precision = 0.6 m (0.2–1.4); Lake2: data yield = 74.7% (50.0–92.9), accuracy = 0.9 m (0.6–1.3), precision = 0.1 m (0.0–0.4)). Vegetated habitat structure and increasing structural habitat complexity generally reduced system performance and added bias (Fig 3; Table 2). For instance, in both lakes data yield dropped from over 70% in the deep open water habitat to close to zero or even zero in dense emergent macrophyte cover. Likewise, accuracy and precision dropped in Lake1 from 1.9 m and 0.6 m in deep open water to 10.3 m and 3.9 m in loose emergent vegetation in Lake1. Corresponding values for Lake2 were 0.9 m and 0.1 m in open water and 1.8 m and 0.6 m in loose emergent vegetation, respectively (Table 2).

**Table 2:** System performance.

**Lake1**							
**Habitat type**	**N**_**Trial**_	**Efficiency (%)**		**Accuracy (m)**		**Precision (m)**	
SubmDense (SD)	4/15	0.7	(0.0–8.2)	6.1	(1.8–20.6)	0.8	(0.0–6.8)
SubmAbove (SA)	15/15	41.6	(12.5–74.5)	4.4	(2.0–10.0)	1.5	(0.4–4.8)
OpenShallow (OS)	45/45	58.1	(31.2–82.7)	3.1	(1.1–9.1)	1.1	(0.2–3.9)
OpenDeep (OD)	50/50	70.8	(49.3–88.4)	1.9	(1.2–3.0)	0.6	(0.2–1.4)
EmerLoose (EL)	14/15	11.6	(1.0–31.6)	10.3	(2.6–40.3)	3.9	(0.3–39.2)
EmerDense (ED)	1/15	0.0	(0–0.1)	-	-	-	-
Overall	129/155	40.0	(6.0–81.1)	3.1	(1.1–8.7)	1.1	(0.2–4.2)
**Lake2**							
**Habitat type**	**N**_**Trial**_	**Efficiency (%)**		**Accuracy (m)**		**Precision (m)**	
OpenShallow (OS)	38/38	76.5	(52.2–94.0)	1.0	(0.6–1.7)	0.1	(0.0–0.5)
OpenDeep (OD)	57/57	74.7	(50.0–92.9)	0.9	(0.6–1.3)	0.1	(0.0–0.4)
EmerLoose (EL)	14/14	24.7	(6.7–49.2)	1.8	(0.6–5.2)	0.6	(0.1–2.7)
EmerDense (ED)	5/14	1.2	(0.0–13.0)	2.3	(1.1–4.7)	1.6	(0.7–3.4)
Overall	114/123	59.8	(20.6–92.7)	1.0	(0.6–1.9)	0.2	(0.0–0.7)

System performance parameters obtained from the linear models (LM; back transformed). Numbers in brackets are the coefficient estimates minus and plus standard deviation which is non-symmetrical due to the back-transformation. N_Trial_ indicates number of trials yielding positions / number of trials in each habitat type.

**Fig 4:**
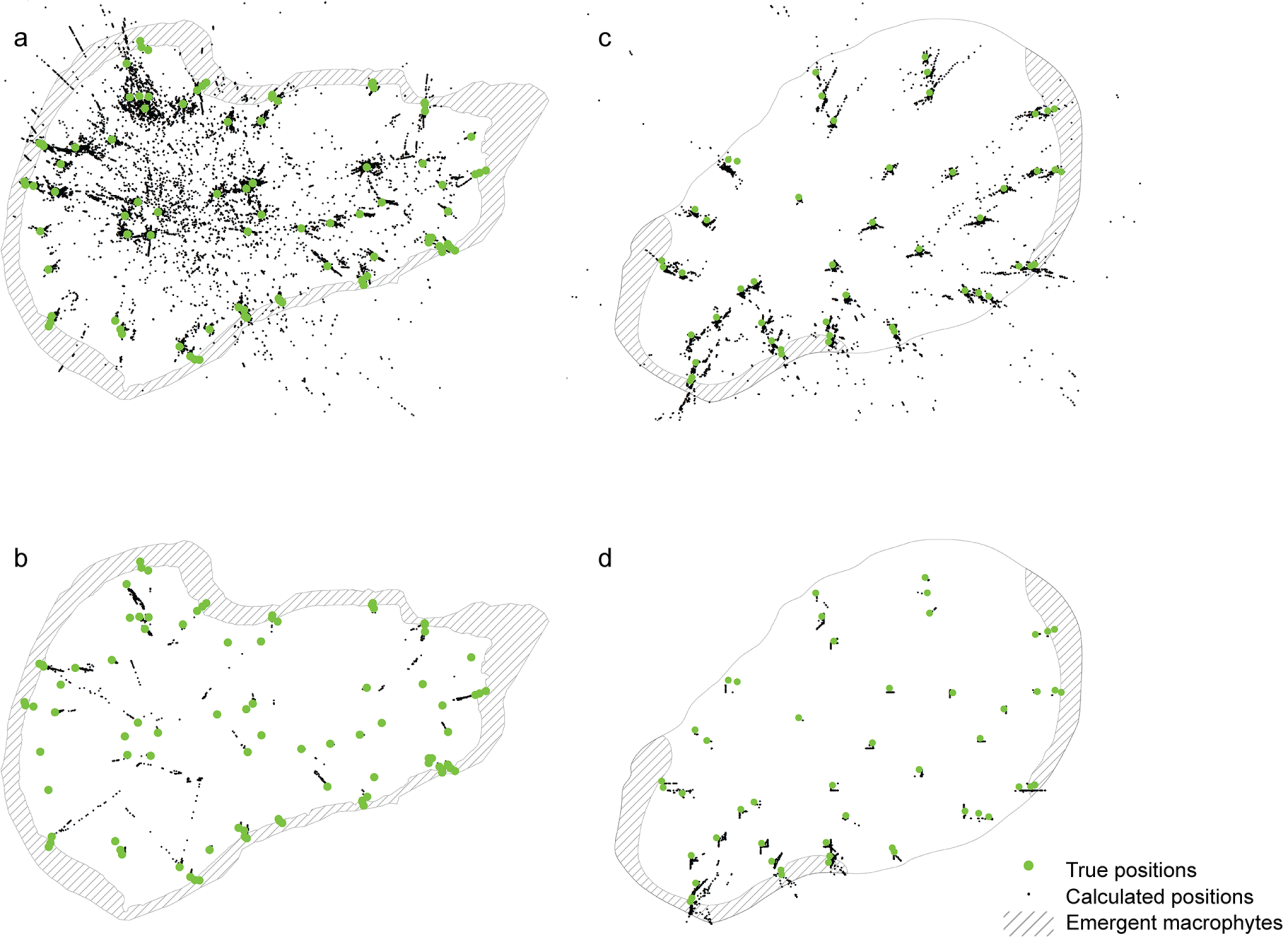
Calculated positions. Calculated positions from the stationary tests before (raw data) and after (filtered data) applying the Hidden Markov Model. Green points indicate true positions of the stationary tests (N_Lake1_: 90; N_Lake2_: 50) and black dots indicate calculated positions. a: Lake1, raw data (N = 54.653); b: Lake1, filtered data (N = 52,015); c: Lake2, raw data (N = 44.024); d: Lake2, filtered data (N = 43,676).

**Fig 5:**
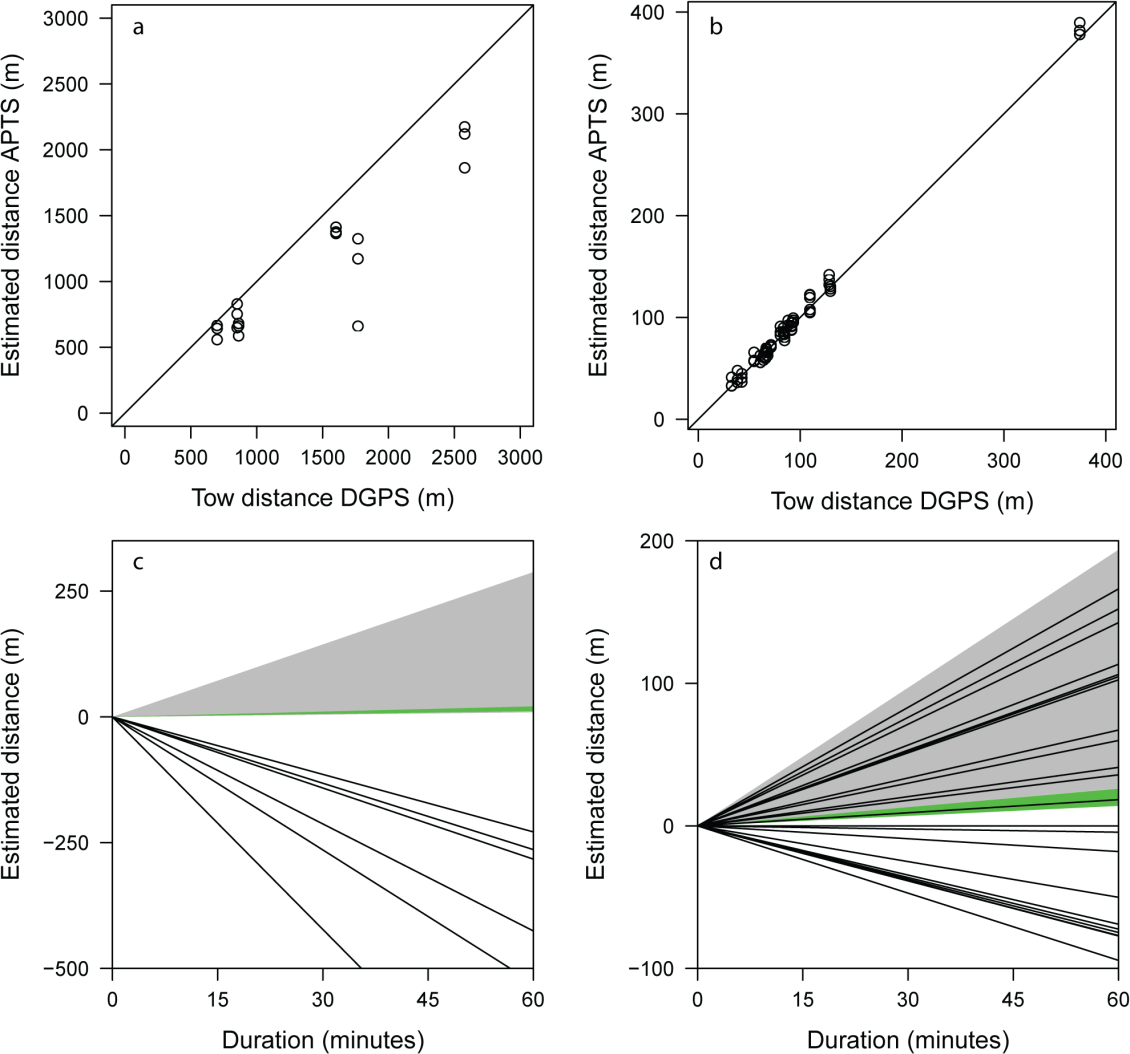
Tow tracks and false movement. *Top row*: Total distance of tow tracks estimated by the APTS versus true distance obtained by DGPS (a: Lake1, n = 18 trials (6 tows); b: Lake2, n = 66 trials (22 tows)). Straight line is the 1:1 iso-line added for comparison, i.e., points below and over this line indicate under- and overestimation, respectively, of track length. *Bottom row*: Accumulated false movement over a 60 minutes period. Filled areas show expected ranges of false movement based on the stationary tests in the open deep water habitat (green; note this area is quite small in both c and d) and the emergent loose habitat (grey). Straight lines represent mean over- or underestimation of track length of each tow (c: Lake1, n = 6 tows; d: Lake2, n = 22 tows).

The processing step involving application of the HMM substantially improved both accuracy and precision compared to the raw data (Figs 3 and 4). For instance, in Lake1 median accuracy and precision of all trials combined improved from 12.1 m and 40.5 m in the raw data to 2.2 m and 0.6 m in filtered data (Figs 3 and 4). Additionally, the filtering step preceding the HMM application resulted in a reduction in system efficiency from an average data yield of 56.7% in the raw data to an average of 50.4% in the filtered data in Lake1 (Figs 3 and 4). Habitat specific mean apparent movement rates (i.e. false movement) ranged from 0.09–4.45 m per position in the various habitats in Lake1 and from 0.02–0.67 m per position in Lake2 (Table 3). Apparent movement of stationary transmitters was generally low with sub-meter bias per position in open water habitats and increased with structural habitat complexity (Table 3). Extrapolation of this bias accounting for position solutions and per position bias led to a modest overestimation of moved distances for stationary transmitters in open water habitats and a substantial overestimation of movement for transmitters in loose emergent macrophytes (Fig 5C and 5D).

**Table 3:** Apparent movement.

**Lake1**			
**Habitat type**	**N**_**Trial**_	False movement (m * pos ^**-1**^ **)**	
SubmDense (SD)	4/15	0.60	(0.79)
SubmAbove (SA)	15/15	0.78	(2.33)
OpenShallow (OS)	45/45	0.27	(0.53)
OpenDeep (OD)	50/50	0.09	(0.24)
EmerLoose (EL)	14/15	3.49	(7.86)
EmerDense (ED)	1/15	4.45	-
**Lake2**			
**Habitat type**	**N**_**Trial**_	False movement (m * pos ^**-1**^ **)**	
OpenShallow (OS)	38/38	0.04	(0.07)
OpenDeep (OD)	57/57	0.02	(0.03)
EmerLoose (EL)	14/14	0.28	(0.31)
EmerDense (ED)	5/14	0.67	(0.33)

Apparent movement per obtained position calculated as average of trial means grouped by habitat category. Standard deviation of trial means are given in brackets. N_Trial_ indicates number of trials yielding positions / number of trials in each habitat type.

### Tow tests

**Fig 6:**
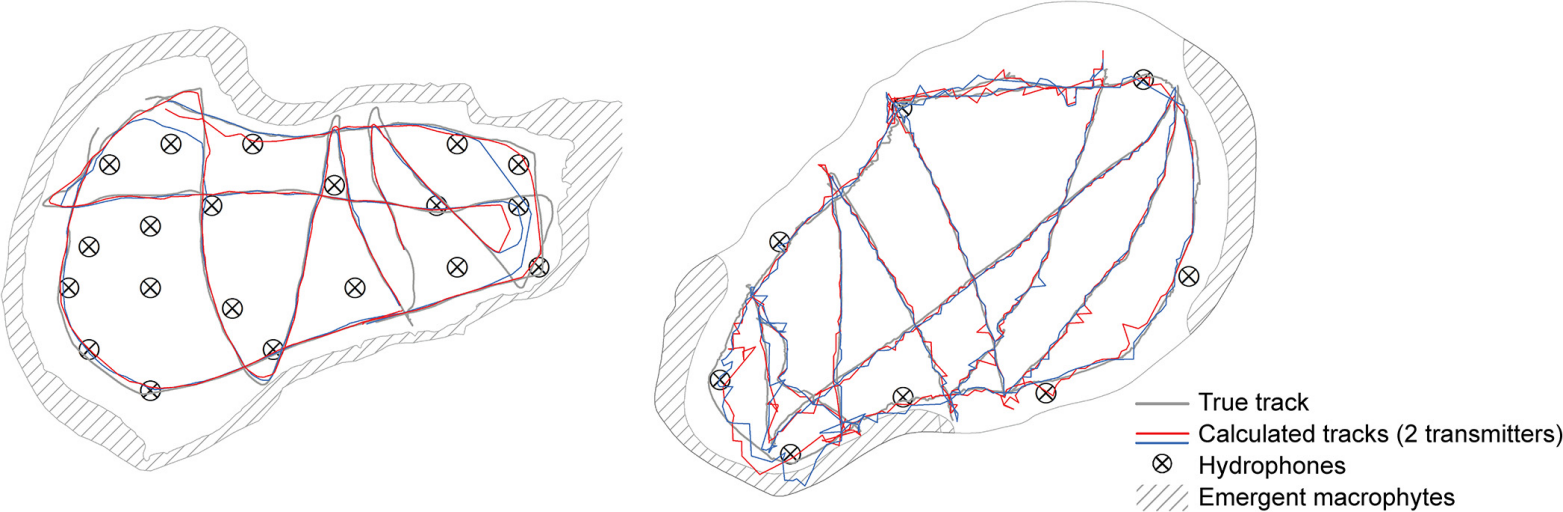
Tow tests visualized. Visualisation of a subsample of the tow tests conducted in Lake1 (left) and Lake2 (right). Tracks from two transmitters in each lake are shown. Overall there were good concordance between calculated (blue and red line) and true trajectory (grey line) in both lakes. However, system performance declined when the transmitters were outside the footprint of the hydrophone array as evident in the western end of both lakes.

Qualitatively deduced by eye, the towed test transmitters yielded a very good fit between the estimated positions and the DGPS trajectory, and hence moving fish would be well tracked by both systems (Fig 6). Mean trial efficiency of the tow tests was 51% (s.d. = 18) and 86% (s.d. = 11) in Lake1 and Lake2, respectively. Corresponding mean accuracy and precision were Lake1: accuracy = 5.3 m (s.d. = 4.0), precision = 7.1 m (s.d. = 5.2); Lake2: accuracy = 0.4 m (s.d. = 0.2), precision = 0.4 m (s.d. = 0.2). Note that the accuracy declined when the towed transmitters were outside the hydrophone array footprint (Fig 6). Total length of the tow tracks in Lake1 were underestimated in all cases whereas both under- and overestimation was evident in Lake2 (Fig 5A and 5B). All presented data and analyses of the tow tracks were based on filtered positions.

### Depth sensor performance

**Fig 7:**
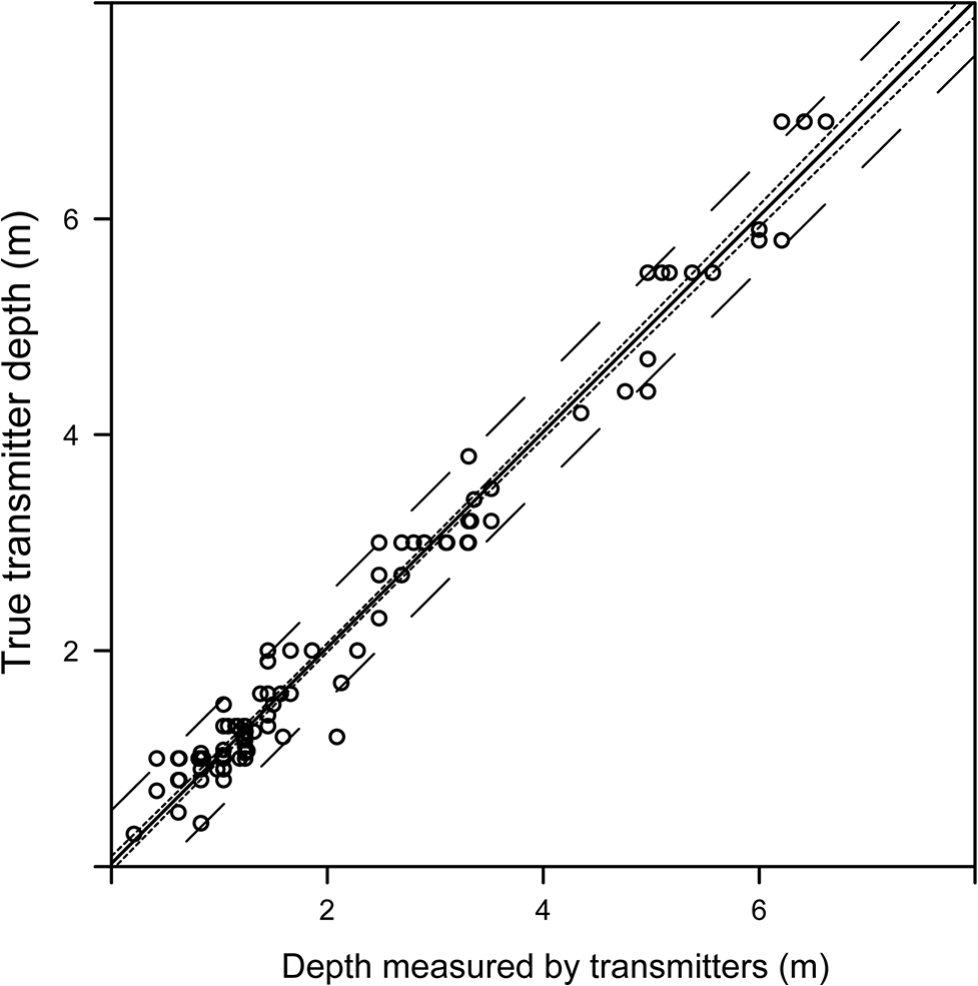
Depth sensor performance in Lake1. True depth measured in the field versus depth estimated from pressure sensors in the stationary transmitters. Solid line is the regression line, broken and dotted lines are 95% confidence and predictions intervals, respectively.

There was a strong linear relationship between transmitter depth and depth reported by the transmitter (Fig 7; n = 155, LM, no intercept, coefficient estimate = 1.01, std. error = 7.5 × 10^–3^, p < 0.0001, adjusted R^2^ = 0.99). Furthermore, the within-trial variation was small (mean s.d. = 0.06 m). These findings indicate that the depths reported by the transmitters were consistently accurate and precise.

## Discussion

We evaluated the performance of two different APT systems covering two lakes differing in habitat complexity and structure, and assessed efficiency (data yield), accuracy and precision of positional data. In both lakes, system performance was high, but it was strongly dependent on structural habitat complexity. Decreasing performance was revealed in structurally complex habitats in all three performance metrics. By the same token, we found both systems to perform very well for tests conducted in the pelagic zone and for tow tracks. In the pelagic areas, accuracy and precision around and below two meters can be expected under favourable acoustic conditions in eutrophic lake ecosystems, and mean accuracy in the open water of Lake2 even reached on average sub meter values. It is unclear whether the better performance in Lake2 was due to the cabled version, size of study lake, hydrophone density, array configuration, transmitter type or due to the particular acoustic environment. Irrespective of the exact mechanism, our results showed that APT systems can generally be recommended for use in lake ecosystems as long as system limitations are taken into account and ecosystem-specific calibration tests are conducted. Our study results are however confined to the particular acoustic situations, including thermal conditions, present in our study sites during the deployment tests as changing environmental conditions in different ecosystems and over time may affect system performance [35,40].

Previous studies utilizing CDMA-based APT systems have often claimed ‘sub-meter accuracy’ in field applications, either with reference to studies by [22] or [23] (e.g., [29,52,53]), own observations [42] or without any supporting reference or study [54,55]. Earlier studies have also documented that sub-meter accuracy is achievable using cabled versions of CDMA-based APT [22,23]. However, replication across lakes and with multiple transmitters is important due to site specific acoustic properties and spatial variation in detection probability. The results from the present study covering two entire two lakes support earlier findings that sub-meter accuracy (and precision) is possible in cabled CDMA-based APT systems. However, our work also revealed that this is not unambiguously true and most likely only holds true in structurally simple pelagic areas.

Raw positions produced by the Lotek software included outliers and even grossly false positions (Fig 4), resulting in poor accuracy and precision of stationary transmitters in both lakes, even after filtering data using the proprietary quality metrics (DOP, RN and CN). Additionally, the Partial Symbol Reconstruction (PSR) engine that Lotek Wireless provides in their positioning software to increase data yield [26] has been found to substantially add errors (including the generation of data when the tag was not in the water [44] and was therefore not employed in the present study. Following the application of a Hidden Markov Model [46,47], system performance improved substantially, confirming that this approach provides a suitable tool to generate high quality positional data yielded by APT systems. However, after applying the HMM, the estimated positions still contained scattering around the true positions. This scatter would induce apparent movement of stationary fish, which can lead to biased estimates of fish swimming activity usually overestimating activity in stationary fish (Fig 5). In field applications, the problem of false movement will increase with the burst rate of the transmitter and the position solution rates (i.e., number of positions) and will be higher for stationary than for mobile fish. This relates to a fundamental trade-off between number of positions that are generated to prove insights into what fish do at all times and certain inferred movement metrics (e.g., total distance swum per day) in high resolution positional telemetry studies. The effect of structural complexity on false movement closely followed the general pattern found in the other performance metrics: best performance (lowest bias) was achieved in open unstructured habitats and decreasing performance as habitat complexity increased.

The results from the tow tests indicated that the APT systems performed equally well for both stationary and moving transmitters. Furthermore, the results illustrated the expected effect of hydrophone geometry on position accuracy, e.g., degradation of accuracy when transmitters were moved outside the hydrophone array. Our findings agreed with earlier work [23] who reported that towed transmitters were well tracked by a cabled APT system in a small Canadian lake. This finding is of crucial importance for the applicability of the systems to successfully study free ranging animals. In this context, the strong linear relationship between transmitter depth and transmitter depth reported by the transmitter indicates that 3D telemetry at high resolution is possible with APT systems.

Researchers might be interested in studying the daily swimming activity patterns using APT systems. In this regard, we found for our specific tow tests that total swum distances was consistently being underestimated in Lake1, whereas both under- and overestimation occurred in Lake2 (Fig 5A and 5B). By contrast, summing the positioning errors for a hypothetically non-moving fish over a period of time would inevitably result in overestimation (degree of which depends on e.g., structural complexity) of daily moved distance (Fig 5C and 5D). As evident from Fig 5D, it cannot be concluded that moving fishes’ distances will be systematically underestimated as this will be highly dependent on several factors including transmitter burst interval, system efficiency, swimming speed and tortuousness of the trajectory. We did not systematically test various movement patterns and speeds, but it is very reasonable to assume that high data yield for a slowly moving fish should approach overestimation patterns as revealed by a hypothetical distance estimate for a stationary fish in Fig 5. The key message here is that while the per position error will be largely independent of how a fish moves, the summation of the errors for behavioural metrics like total distances moved will contribute to a pattern of movement type-dependent biases in total distances.

Our findings reflect the complex nature of aquatic acoustic positional telemetry. In general, the probability of detecting a signal at a given hydrophone is a function of the amount of signal attenuation through obstacles such as macrophytes, and signal interference from multipath propagation caused by signal reflection from hard substrates or the water surface (e.g., [40,56]). Furthermore, in APT an emitted signal needs to be detected by at least three hydrophones in a suitable geometry to facilitate a high quality position solution. Because signal attenuation is positively correlated with structural habitat complexity, the observed decrease in system efficiency in structured habitats in our study was expected, but the magnitude has not previously been quantified.

The between-habitat variation in both accuracy and precision reported here can partly be explained by geometric issues inherent in the positioning algorithm [22]. Generally, the performance of the algorithm based on hyperbolic trilateration peaks at positions within the centre area of an equilateral triangle outlined by three hydrophones and degrades gradually for positions closer to and outside the triangle edges. Thus, positions outside the core hydrophone array will generally be less accurate than positions inside it [22,25,26,38,57]. In Lake1, littoral habitats, which also happened to be vegetation-rich (leading to vegetation and geometry being confounded), were usually located outside the main hydrophone array. Hence, erosion of accuracy and precision (but not data yield) in littoral zones can be expected for geometry reasons unrelated to vegetation. Alternatively, as elaborated above it is conceivable that complex structured habitats can cause more signal interference than non-structured open water habitats; therefore, the habitat type *per se* might also influence system accuracy and precision. Further insights could potentially be gained by studying the between-tag performance variation of co-located and co-towed transmitters, but this is beyond the scope of the present study. Irrespective of the exact mechanisms, claims for sub-meter accuracy of APT systems primarily hold for ideal acoustic conditions, such as those present in noise and obstacle free open water habitats inside footprints of hydrophone arrays and are unlikely to hold for vegetation-rich littoral zones where optimal geometries for positioning cannot easily be achieved.

The APT in Lake2 performed better than the APT in Lake1. This was most likely related to differences in lake size and hydrophone array configuration and may have been facilitated by the use of a continuously clock-synchronized cabled APT system deployed in Lake2. Lake1 was approximately 20 times larger than Lake2 and, although more hydrophones were deployed in Lake1 to accommodate limits in acoustic range and signal blocking caused by vegetated habitat structure, the coverage of Lake1 in terms of between-hydrophone distances and area outside the hydrophone array was coarser. Furthermore, the proprietary positioning algorithm only allowed utilizing a maximum of eight hydrophones simultaneously. To achieve coverage of the whole Lake1, we defined 13 separate 8-hydrophone arrays, which traded off the need to cover the whole lake and increased computational time needed to position and post-process data. Although the approach applied in Lake1 (defining several sub-arrays) can remedy some of the limitations of the commercial positioning software, researchers should be aware of many challenges that emerge from it, in particular in relation to the number of arrays to be tested and the increase in computational effort. Additionally, emitted signals by a given transmitter were occasionally detected by two or more sub-arrays resulting in multiple possible positions originating from a single emitted signal. It is not immediately obvious in a field application which of these positions is the most accurate. Finally, the inevitable time drift of the internal clocks in the wireless hydrophones causes de-synchronization of the defined sub-arrays and lead to different time stamps on the multiple positions originating from a single signal, which needs to be addressed using further assumption at the analysis stage.

### Conclusions and Recommendations

We conclude that both CDMA-based APT systems are capable of providing positional data of high quality in terms of both temporal and spatial resolution at the ecosystem level. We therefore claim that the CDMA-based APT systems are indeed potent tools for mining the reality [12] of aquatic animals, in particular in relation to swimming activity, space use, social behaviour and choice of habitats. Such systems offer a major improvement to traditional telemetry methods based on manual tracking or presence/absence hydrophone curtains. However, it should be noted that sub-meter accuracy is not to be expected throughout an entire study site and can actually be the exception in some lakes. Additionally, limitations are to be expected within complex habitat structures because efficiency, accuracy and precision decrease in these structures, and tracking error will lead to false apparent movement. Nevertheless, by deploying an APT to cover an entire ecosystem, it is possible to turn a natural lake into a field-laboratory providing detailed behavioural information of tagged animals.

For future studies, the effects of structural complexity and hydrophone geometry on system performance should be considered during the design and analysis phases. For instance, focal species that are known to reside in complex habitats close to the shore might warrant a different hydrophone array configuration than pelagic species primarily residing in the centre of a lake in order to facilitate optimal coverage. We envision APTs to be best suited for studies based on activity, studies of animal social systems based on proximity measures and manipulative experiments. However, as every lake is unique in terms of bathymetry and acoustic properties, we strongly advocate that rigorous tests of system performance are conducted as an integral part of the initial phases of studies using APT systems. Furthermore, knowledge of the spatial variability of system performance will facilitate the use of statistical models incorporating this variability in the biological analyses. Additionally, we suggest placing a number of stationary transmitters in fixed positions for the duration of any study to be able to quantify and account for potential temporal variation in system performance [58]. Without proper knowledge on the performance of a particular system setup, erroneous interpretations of data and, subsequently, false conclusions are likely to be made.

The application of a HHM improved system performance in terms of both accuracy and precision. Therefore, researchers are advised to consider the possibility of using such statistical options if high spatial resolutions are needed for answering specific research questions. Additionally, we advise researchers to ensure that they have access to the know-how required to process raw data as well as data handling and analyses of the potentially very large data sets obtained using APT systems. This includes the development of databases and analysis algorithms, which, to a large extent, will need to be custom designed for each specific research project. Note that processing time of both systems is substantial and involves a considerable amount of manpower. For example, processing one month of field data can take several days or weeks of post-processing, data cleaning and modelling depending on system configuration and number of tagged animals. Additionally, the high costs per transmitter will limit the number of animals that typical research projects will release. Nevertheless, a new toolbox is available allowing high resolution, fine scale behavioural studies at the scale of entire ecosystems thereby facilitating reality-mining of free ranging aquatic animals.
